# Advanced Vaccine Candidates for Lassa Fever

**DOI:** 10.3390/v4112514

**Published:** 2012-10-29

**Authors:** Igor S. Lukashevich

**Affiliations:** Department of Pharmacology and Toxicology, School of Medicine, and the Center for Predictive Medicine for Biodefense and Emerging Infectious Diseases, University of Louisville, Kentucky, USA; Email: isluka01@louisville.edu; Phone: +1-502-852-8822; Fax: +1-852-5468.

**Keywords:** Lassa fever, pathogenesis, vaccines, efficacy in primates

## Abstract

Lassa virus (LASV) is the most prominent human pathogen of the *Arenaviridae*. The virus is transmitted to humans by a rodent reservoir, *Mastomys natalensis*, and is capable of causing lethal Lassa Fever (LF). LASV has the highest human impact of any of the viral hemorrhagic fevers (with the exception of Dengue Fever) with an estimated several hundred thousand infections annually, resulting in thousands of deaths in Western Africa. The sizeable disease burden, numerous imported cases of LF in non-endemic countries, and the possibility that LASV can be used as an agent of biological warfare make a strong case for vaccine development. Presently there is no licensed vaccine against LF or approved treatment. Recently, several promising vaccine candidates have been developed which can potentially target different groups at risk. The purpose of this manuscript is to review the LASV pathogenesis and immune mechanisms involved in protection. The current status of pre-clinical development of the advanced vaccine candidates that have been tested in non-human primates will be discussed. Major scientific, manufacturing, and regulatory challenges will also be considered.

## 1. Introduction

Lassa virus (LASV) is transmitted to humans by a rodent reservoir, *Mastomys natalensis,* and is capable of causing lethal Lassa Fever (LF) disease. There is no licensed vaccine for the prevention of LF and vaccine development efforts are hampered by both the high cost of non-human primate (NHP) animal models and biocontainment requirements (BSL-4) for study and development. LASV has the highest human impact of any of the hemorrhagic fever viruses (with the exception of Dengue fever) with an estimated 100,000-300,000 infections and 5,000-10,000 deaths annually in western Africa [1,2,3,4,5]. Based on prospective studies performed in four of the most affected countries, Guinea, Sierra-Leone, Liberia, and Nigeria, Richmond and Baglole [5] estimated that 59 million people are at risk of primary LASV infections with an annual incidence of disease as high as 3 million and as many as 67,000 deaths per year. The current LF predicted areas (Figure 1A) cover approximately 80% of Sierra-Leone and Liberia, 50% of Guinea, 40% of Nigeria, 30% of each of Côte d’Ivoire, Togo and Benin, and 10% of Ghana [3]. LASV was also detected in Mali [6] and LASV antibodies were detected in the Central African Republic, Democratic Republic of Congo, Senegal. Some experts believe that the population at risk includes most of the population of West Africa from Senegal to Nigeria and can be high as 200 million [5]. Recently performed genome-wide scans [7,8] suggest that LASV (and/or LASV-like viruses) may have been a driver of natural selection of genes implicated in LASV infectivity and immune responses in West African population.

Given the high annual incidence rate and mortality, it is arguable that LF is one of the most neglected tropical diseases, to the point that some have pointed out that if LF was a Developed World problem, there would be vociferous demands for control measures and vaccine [9]. An effective LASV vaccine is urgently needed not only for the general population, but also for healthcare and lab workers, as well as for military and other service personnel in West Africa. The vaccination strategies may differ for the various recipient populations. Whereas a multi-dose immunization regimen might be practical for medical providers and for military personnel, a single-dose vaccine would be ideal in endemic areas, where most of the target population is poor and live far from health care facilities [9].

**Figure 1 (a):**
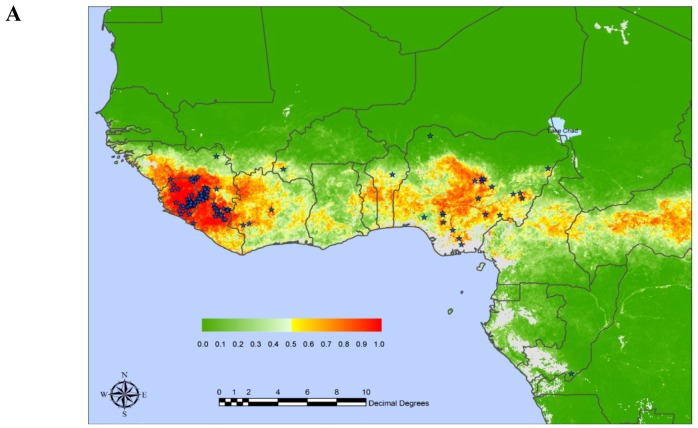
Risk map of Lassa Fever in West Africa. Positive localities indicated by stars. The posterior probability color scale, from 0.0 (no risk) to 1.0 (highest risk) is shown as an inset. From E. Fichet-Calvet, D.J. Rogers [3] with permission.

**Figure 1 (b):**
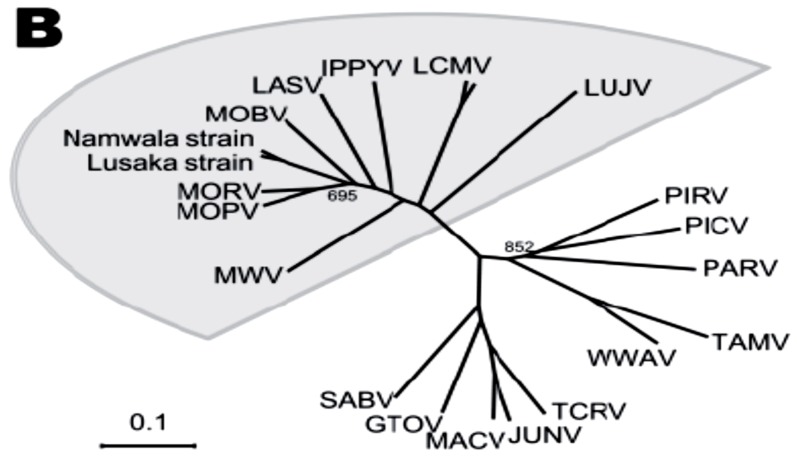
Phylogenetic relationships among the Old World arenaviruses (grey sector) based on analysis of the NP gene. From A. Ishii et al with permission [10]. See text for more details.

A cell-mediated immunity (CMI) plays the major role in the recovery and prevention (see below) and “a single shot of live attenuated candidate vaccine conferring life-long immunity is much preferred” [9]. In this review the current status of vaccine approaches to control LF will be overviewed with major attention to vaccine candidates tested in NHP models. Obstacles and challenges on the route of these vaccines into early stage of clinical development will be discussed as well.

## 2. Lassa Virus Diversity and Novel African Arenaviruses

Lassa virus (LASV) belongs to the *Arenaviridae,* a fast-growing family of rodent-borne viruses, currently including two dozen envelope viruses with bi-segmented, ambisense single-stranded RNA genomes [11,12]. The large (L) genomic segment encodes an RNA-dependent RNA polymerase (RdRp) and a small RING finger Z protein (analogous of matrix protein). The small (S) genomic segment encodes the nucleoprotein, NP, and the glycoprotein precursor, GPC [13]. Based on their antigenic properties and geographic distribution arenaviruses are divided into two complexes, the Old World (OW) arenaviruses circulated in Africa (Figure 1B) and the New World (NW) arenavirus circulated in Americas. The prototypic lymphocytic choriomeningitis virus (LCMV) has a global distribution. Currently the OW group, or LCMV-LASV complex (Figure 1B) contains LCMV which can cause neurological pathology in adults, fetal abnormalities in newborns, and a fatal LF-like disease in immunocompromised patients; LASV; and non-pathogenic viruses, Mopeia (MOPV), Morogo (MORV), Mobala (MOBV), and Ippy (IPPYV) [14]. Several new viruses recently isolated in Africa rapidly expended this group (see below). The NW arenaviruses are divided into three major clades, A, B, and C with Clade B containing Junin (JUNV), Machupo (MACV), Guanarito (GTOV), Sabia (SABV), and Chapare (CHPV) viruses associated with severe hemorrhagic fevers in South America [15,16]. Recombination of GPC sequence was found in North American arenaviruses, Tamiami (TAMV), Whitewater Arroyo (WWAV), and Bear Canyon (BCNV), and these viruses were placed into a separate lineage named A/Rec [16,17].

Genetic diversity among LASV strains is the highest among the *Arenaviridae*, and causes a great challenge for vaccine development. Based on the partial NP sequences of 54 strains of LASV, Bowen et al. [18] showed that LASV isolates comprise four lineages, three of which are found in Nigeria, with the fourth found in Guinea, Liberia, and Sierra Leone. This diversity even raised concern about the status of LASV as a single species [18,19,20]. The prototype LP strain isolated by Buckley & Casals in 1969 from Eastern Nigeria occupied the most basal lineage I. Strains isolated from Southern Central and Northern Central Nigeria were placed in lineage II and III, respectively, and the largest group of strains from Guinea, Liberia, and Sierra Leone occupied lineage IV are sufficiently related to suggest that Nigerian strains from lineages I and II diverged prior to strains from northern part of central Nigeria and Guinea, Liberia, and Sierra Leone. A fifth lineage, which falls between III and IV, has been proposed for the AV strain isolated from a patient that was infected (presumably) in Ghana or Ivory Coast [21]. 

Overall strain variation among the 54 strains is as high as 27% and 15% at the nucleotide and amino acid levels, respectively. The genetic distance between different strains correlates with geographic distance, rather than time of strain isolation, with no evidence of a “molecular clock” to be found [18]. Full-sequence analysis did not find evidence of recombination among LASV strains and showed that Nigerian LASV strains seem to be ancestral to strains from Guinea, Liberia, and Sierra Leone. Surprisingly, this analysis also demonstrated that structural genes in the S RNA segment had a lower variability in comparison with the polymerase gene, RdRp [18,22,23]. Analysis of evolutionary history of LASV suggests that the virus appeared 750-900 years in Nigeria and has been relatively recently moved into the western endemic areas [18,24,25]. This westward movement across West Africa seems to be accompanied by some levels of attenuation of virus pathogenesis. Indeed, it has been noted that case fatality rates in the western endemic areas are lower than in Nigeria [26].

In addition to LASV, other African arenaviruses that are not pathogenic for human and NHP [27] include: IPPYV [28,29], isolated from *Arvicantis* spp; MOBV [30] isolated from *Praomys* spp. in the Central African Republic (CAR), and MOPV isolated from Mozambique [31], and Zimbabwe [32]. Although antibodies against MOPV were detected in people [33], there is no evidence of clinically-manifested LF in areas of MOPV circulation. These observations taken together with the ability of MOPV to protect NHP against LASV challenge [34,35] allow some researchers to consider MOPV a naturally attenuated variant of LASV [36].

Both pathogenic LASV and non-pathogenic MOPV are associated with the poorly defined *Mastomys *spp. [37]. The proportion of infected *M. natalensis* can vary considerably, even between villages in close proximity, and can be as high as 30% [38]. Infected rodents tended to cluster in relatively few houses, suggesting the existence of focal "hot spots" of LASV-infected rodents, which may account for the observed heterogeneous distribution of LF [39].

Little is known about impact of LASV persistent infection on the eco-epidemiology of *Mastomys *in West Africa and available information is contradictory [39,40]. Effect of LASV persistent infection on fitness of *M. natalensis* and on a commensal lifestyle in West Africa has to be further investigated. As it has been shown recently, this fitness can be affected by anthropogenic factors which can change genetic diversity of the populations of *M. natalensis* and LASV in West Africa [25].

Advanced screening of small mammals in LASV endemic areas provided evidence for existing novel African OW arenaviruses (Figure 1B). Kodoko virus (KODV), which is related to but distinct from LCMV, was isolated in Guinea [41]. In 2008, Lujo virus (LUJV) was isolated in Southern Africa during a nosocomial hemorrhagic fever outbreak with unprecedented high case fatality rate [42,43]. Screenings of *M. natalensis* trapped in Zambia, where the first patient infected with LUJV was identified, found two strains, Lusaka and Namwala, of Luna virus (LUNV) [10] (Figure 1B). Nevertheless, LUJV seems to be a unique virus and branches off the ancestral node of the OW arenaviruses [43]. Genetic and predicted protein analysis suggests that LUNV is more similar to MOBV than to MOPV-MOPV (Figure 1B). Merino Walk virus (MWV), a proposed novel tentative OW species was isolated from a rodent, *Myotomys unisulcatus*, collected at Eastern Cape, South Africa [44]. Analysis of the full-length genomic sequence revealed that MWV is distantly related to MOPV-MORV viruses isolated from *M. natalensis *in Mozambique and Zimbabwe. In Côte d’Ivore, 60% of rodents captured in different geographic areas were identified as *M. natalensis* but these rodents did not harbor LASV. Still, two novel arenavirus sequences were found by advanced RT/PCR; with tentative names Menekre (MENV) and Gbagroube (GBAV) viruses, those sequences were detected in *Hylomyscus sp*. and *Mus (Nannomys) setulosus*, respectively [45]. Notably, GBAV sequence was closely related to LASV, while MENV sequence clustered with IPPYV-MOBV-MOPV. Detection of LASV-like sequences in *Mus setulosus* suggests that co-evolution of African arenaviruses and their hosts can potentially include host-switching events predicting isolation of novel arenavirus species in the future.

Interestingly, a group of novel viruses distantly related to arenaviruses but also to filoviruses, was isolated from snakes with fatal IBD (inclusion body disease) [46]. Isolation of arenaviruses from non-mammal hosts indicates that these viruses can infect very broad range of species with unpredictable pathogenic potential for humans.

Natural infection of *M. natalensis* with genetically closely related arenaviruses suggest that, under some circumstances, these viruses can be involved in recombination or an exchange of entire segment (reassortment), both of which are common mechanisms in the evolution of RNA viruses. Indeed, recent cross-species analysis of the replication complex of LASV, MOPV, and LCMV showed that minigenome (MG), NP, and L protein could be exchanged between the replicon systems of LASV and MOPV without loss of function. However, the LCMV L protein is required the homologous NP for activity in replicon system [47]. These observations are consistent with successful generation of stable MOP/LAS reassortants after co-infection of cell with both viruses [48,49]. In spite of experimental evidence of reassortment between two virus species, LASV and MOPV, there is no evidence for natural reassortment or recombination between two viruses, and it is highly unlikely that reassortment occurred in the past for any currently known arenavirus species [16,22].

## 3. Lassa Fever Pathogenesis and Mechanisms of Protection

As mentioned above, LASV is the most prevalent arenavirus in West Africa. Fortunately, ~ 80% of LASV-infected individual express sub-clinical or flu-like manifestations and the overall case-fatality rate is ~ 1-2%. However, in hospitalized patients and in some risk groups (pregnant women, children <5 years old, immunocompromised individuals, *etc*.) the fatality rate can be higher than 50% [1]. After recovery, in 29% of LF patients, acute manifested infection is accompanied by a sensorineural hearing deficit, which accounts for a permanent hearing loss in 18 % of survivors [50,51].

The pathogenesis of LF is still not clearly understood. Severe LASV infection is characterized by unchecked viremia, functional liver damage, and immunosuppression [1,52]. Host factors such as a cell receptor polymorphism [8], innate immunity [53,54,55,56], pro-inflammatory cyto/chemokines [56,57,58,59], adaptive CMI responses [60,61] as well as differences in pathogenic potential of LASV isolates [62] seem to play a role in outcome of LASV infection in humans. LASV replicates in target tissues (liver, spleen, lymph nodes, adrenal) without cytopathic effect, and the pathological damage to these tissues is usually not sufficient to implicate organ failure as the cause of death [63,64,65]. In fact, death from LF is caused mostly by uncontrolled sepsis-like terminal cardiogenic shock and internal bleeding. In contrast to HFs caused Ebola and Marburg viruses [66], a disseminated intravascular coagulation is not involved in LF pathogenesis, bleeding abnormalities are not common. Indeed, it has been even questioned if LF is pathologically a hemorrhagic fever [63,66].

Natural rodent-to-human LASV transmission occurs via inhalation of contaminated droplets/dust, or through ingestion of contaminated food [1,67]. Respiratory entry of arenaviruses occurs via basolateral receptors [68]. Recently we have shown that the viruses enter polarized Caco-2 cells from the basolateral site as well (Lukashevich et al; unpublished). These observations are in good confirmation with basolateral location of major LASV cell receptor, α-dystroglycan (αDG), and with general perception that basolateral entry provides easiest way to access target cells, macrophages and DCs. While entry requires the basolateral surface, LASV can be released from the apical cell surface. At least in polarized kidney-derived cells (MDCK), LASV virus-like particles (VLPs) exit cells from the apical site and this process was driven by GP [69]. Excretion of LASV from kidney of persistently infected *M. natalensis* is the major source of natural contamination for human exposure.

In spite of recent success in our understanding of LASV-host receptor interaction (rev. [70]), the detailed mechanism of LASV entry is unclear and the question remains how the virus crosses through respiratory and gastro-intestinal epithelia. LASV is remarkably resistant to low pH [71]. This factor combined with our epidemiological observations in Guinea, where hunting of peridomestic rodents and consumption of their meat are possible risk factors [67], suggest that gastric and/or intestinal epithelial cells can be the major gait entry. This hypothesis is supported by data from experimentally infected (through gastric rote) rhesus macaques [72]. The requirement of the virus for basolateral entry suggests that integrity of the epithelia must be compromised for the virus to get access to the basolateral site. However, as LASV infection is not cytopathic to epithelial [68,69] and endothelial [57] cells and does not appear to alter barrier function [69], external factors affecting epithelia integrity may contribute to the infection.

The virulence of LASV in experimentally infected animals is directly related to virus replication, and it is not related to classic T cell-mediated pathology (rev. [73,74]). Clinical studies showed that high viremia (≥ 1 x 10^3^ TCID_50_/ml) was associated with high fatality rate and elevated viremia together with a high aspartate aminotransferase level (AST ≥ 150 IU/L) carried a risk of death in 78% of the cases. Similar observations were made in the LF model in NHP [27,72,74,75,76,77,78]. Based on data available so far, the major events leading to fatal outcome are summarized below: (i) from the initial sites of infection the virus spreads to regional lymph nodes, liver, spleen and infects tissue macrophages and DCs; (ii) release of cyto/chemokines from these cells recruits additional macrophages to sites of infection, amplifies deregulated host responses, contributes to loss of lymphocytes, down-regulates their functions; (iii) failure to properly initiate innate and adaptive immune response results in ineffective virus control; (iv) pro-inflammatory mediators from infected macrophages and DCs affect endothelial function and virus-induced impairment of vascular functions contributes to edema and shock at terminal stages; (v) infection of Kupffer cells (KCs), hepatocytes, and adrenal cortical cells additionally contributes to the hemodynamic and coagulation dysfunctions and results in hypotension and sodium loss with hypovolemia. Combined together, these events lead to sepsis-like shock leading to fatal outcome [2,52,66,79].

The production of pro-inflammatory cytokines results in the activation of antigen-presenting cells and T cell stimulation in the adaptive immune response and is required for clearance of viral infection. A large body of evidence accumulated during the last decade indicates that LASV-induced immunosuppression contributes to fatal outcome. We previously showed that replication of the OW pathogenic arenaviruses (LASV and LCMV-WE), but not non-pathogenic (MOPV and LCMV-ARM), was associated with the inhibition of pro-inflammatory cyto/chemokine responses *in vitro* and *in vivo* [57,80,81]. Indeed, the cytokine responses in LF patients and in experimentally infected NHP showed no evidence for a “cytokine storm”, which was observed in filovirus-induced hemorrhagic fevers (rev. [52]). In the progressed LF patients, fatal infection correlated with low or undetectable levels of pro-inflammatory cyto/chemokine [58]. At present, the immunosuppressive phenotype of fatal LASV infection is a well-accepted concept [52,82,83,84,85]. In human DCs-T-cell co-cultures, LASV induced only weak memory phenotype markers, while MOPV strongly stimulated CD8+ and CD4+ T cells, activation markers, proliferative responses, and CTL activities [83]. We have also shown recently that pathogenic arenaviruses, LASV and LCMV-WE, suppress NF-kB-mediated TLR2/Mal-dependent pro-inflammatory cytokine responses in infected monocytic cells [56]. In contrast, MOPV induced strong up-regulation of cytokines known to contribute to the development of robust adaptive immune responses. Similarly, in LCMV-ARM-infected mice, TLR2/MyD88/Mal signaling played an essential role in antiviral CD8+ T cell responses [86]. In the absence of MyD88, naive CD4+ T cells failed to differentiate into LCMV-specific CD4+ T cells [87]. Whether the blunted induction of cytokines is a direct or indirect outcome of virulent virus infection, the lack of innate stimulation likely positions the host to have a delayed and/or inefficient adaptive immune response to clear the infection. The immunosuppressive phenotype of LASV infection must be taken into consideration during rational vaccine design. An ideal vaccine has to induce strong, long-lasting protective immune responses prior LASV challenge.

The major cellular receptor for LASV, other OW arenaviruses, and Clade C of the NW viruses is α-DG, a cell surface receptor for extracellular matrix (ECM) proteins [88]. In the spleen, over 99% of α-DG is associated with DCs and less than 1% with CD4+ and CD8+ T cells. LASV and LCMV-WE54 (causes a LF-like fatal disease in NHPs) bind to α-DG with high affinity, preferentially infect DCs, effectively compete with and displace ECM molecules of DCs, and alter their ability to initiate an effective immune response [89,90]. In contrast, the low affinity of MOPV and LCMV-ARM (both viruses are not pathogenic for NHP) to α-DG correlates with their ability to induce TLR2/MyD88/Mal-dependent cytokine responses [56] and with the adaptive CMI responses to effectively control virus replication and restrict viral pathogenesis. However, several findings suggested that that α-DG is not the sole receptor for LASV and LCMV [89,91]. In fact, the level of α-DG is the highest in muscle tissues, where the LASV load in minimal. In contrast, the viral burden is very high in hepatocytes during the infection; these cells do not express α-DG. Recently two C-type lectin family members, DC-SIGN and LSECtin, and two TAM family members, Axl and Tyro3, have been recently identified as LASV and LCMV receptors [92,93], which may be responsible for replication in hepatocytes.

Recovery of LF patients and experimentally infected animals was not related to antibody levels and protection of animals in challenge experiments was associated with CMI [27,60,61,94,95,96]. Controlled clinical trials with human convalescent plasma containing high titers of antibodies failed to protect [74]. Although prime-boost immunization of primates with concentrated and gamma-irradiated LASV induced strong antibody responses to NP and GP, the immunized animals were not protected against challenge [97]. In naturally-infected individuals, anti-LASV antibodies had a short life-time and become undetectable in a few months [9,98] . Nevertheless, these “seronegative” individuals had strong CD4+ proliferative responses against LASV NP and GP antigens [60,61]. Importantly, these individuals cannot be protected against re-infection, but they are fully protected against disease indicating that a single primary infection with LASV confers long-term protection against LF [9].

The clearance of LASV does not correlate with antibody induction and LASV is a poorly neutralized virus. Clinical and experimental studies in infected NHPs showed that viremia and circulated antibodies were detected simultaneously in LF patients and infected animals (rev. [1,2,9]). At the time of hospital admission, LASV antibodies against NP or GP were not associated with survival or positive prognosis. Indeed, the presence of LASV antibodies detectable in IFA early in the course of the disease correlated with fatal outcome, not survival [99]. Recent studies confirmed the previous observation and showed that patients simultaneously containing LASV antigen and specific IgM antibodies have significantly higher chances (>4 times) to die in comparison with patients with IgM alone [100]. Taken together these results clearly indicate that protection and recovery are not associated with LASV antibody responses.

## 4. Non-Replication Competent Vaccine Platforms

### 4.1. “Killed” Vaccines and Virus-Like Particles

A favorable safety profile is the most attractive feature of inactivated (‘killed’) vaccines or virus-like particles, but these approaches in general have low immunogenicity and efficacy. To achieve desirable levels of protection these vaccines therefore require multiple prime-boost injections. Although this would be difficult to conduct in rural areas with poor healthcare infrastructures, such as the areas of West Africa where LASV is endemic, our group and others have tested the efficacy of this approach. In our experiments, extensive prime followed by multiple boost i.p. immunizations of CBA/Calac mice with highly purified, gamma-irradiated LASV preps resulted in some levels of protection against i.c. challenge, but this appeared to be mediated mostly by CMI (Godneva & Lukashevich, unpublished). However, although a similar approach in NHP induced excellent antibody responses, to NP and GP antigens as mentioned above, all vaccinated animals died after LASV challenge; postmortem titration did not reveal differences in tissue viral burden between vaccinated and non-vaccinated [97]. There is only one report published two decades ago in the Russian journal “Vopr. Virusol” [101] that describes protection of *Papio hamadryads* immunized with inactivated LASV preps against a subsequent LASV challenge. The authors demonstrated some level of protection against a relatively low challenge dose, 0.4 PFU. These results have not been independently reproduced or confirmed by other groups, and a model of human LF in hamadryads has not been developed.

Recent success of VLP-based vaccines against HPV motivated some researches to also apply this approach to LASV; they produced VLP by a transient expression of LASV GP, NP, and Z genes in mammalian cells [102]. Although some LASV VLP were immunogenic in mice after one prime and two boost immunizations, the VLP preps were contaminated with glycosylated proteins (30 to 220 kDa), presumably acquired during budding from the cell membrane or the Golgi apparatus [102]. Contamination concerns raise questions regarding a feasibility of this technology in manufacturing environment.

It is likely that the absence of viral RNA species in VLP contribute to the low effectiveness of LASV VLP. Specifically, successful viral vaccines (e.g; influenza vaccines) most often contain viral RNA that act as 'built-in' adjuvants. Specific receptors, RIG-I-like, Toll-like, and nucleotide-binding oligomerization domain-like receptors can detect specific nucleic acid patterns at early stages of antiviral responses, engage CMI, and thereby significantly enhance the adaptive immune responses [103,104,105,106,107]. Recent studies showed that RNA isolated from LASV was able to activate the IFN-β promoter [108]. The RNA-inducible TLR7-mediated signaling was required for activation of plasmocytoid DCs at early stages of infection to mount an effective adaptive immune responses that prevented development of persistent arenavirus infection [109,110].

Taken together, although “killed” virus or VLP can induce antibody responses in experimental subjects, the prospect of these approaches translating into an effect vaccine strategy is low. In spite of the fact that the current prospect of developing a killed LASV vaccine is low, novel adjuvant systems (e.g; ISCOMATRIX) [111] which significantly increase both antibody and CMI responses, can provide a new opportunity for a killed vaccine approach.

### 4.2. Peptide epitope-based vaccine approaches

Another potential approach is epitope-based vaccines. Using computer-assisted algorithms, five HLA-A2.1-binding LASV GP peptides and two LASV NP peptides have been identified [112,113]. When HLA-A*0201 transgenic mice were immunized with either LASV peptides GPC(42-50) or GPC(60-68), they were indeed protected against a subsequent challenge with a recombinant vaccinia virus that expressed LASV GPC [113]. In the recent study [114], a panel of HLA-A*0201-restricted peptides derived from the same region of the LASV GPC(441-449), LCMV GPC(447-455), JUNV GPC(429-437), MACV GPC(444-452), GTOV GPC(427-435), and WWAV GPC(428-436) was identified that displayed high-affinity binding to HLA-A*0201; and these peptides were cross-reactively recognized by CD8+T cells following LCMV infection or peptide immunization in HLA-A*0201 transgenic mice. Immunization of HLA-A*0201 mice with LASV GPC(441-449) or LCMV GPC(447-455) induced high-avidity CD8+- T-cell responses that were able to kill syngeneic target cells pulsed with either LASV GPC(441-449) or LCMV GPC(447-455) in vivo and provided significant protection against viral challenge with LCMV [114].

Although the peptide-based approach looks promising, especially for high-risk infectious agents, peptide vaccination is unlikely to be applicable for LASV because of significant safety concerns. As has been discussed, CMI responses, in particularly CD8+ T cells, play the major role to control primary infection; the CD8+ memory cell progeny also play a major role in protecting against subsequent re-infection. In addition to directly destroying infected cells, CTL (cytotoxic T lymphocytes) also release potentially cytotoxic cytokines (e.g; IFN-γ and TNF-α) that result in some degree of immunopathology. In fact, some of the systemic symptoms of viral infections are caused immune responses rather than by the infection itself. Although the precursor frequency of naïve T cells specific to any give epitope is low ~ 1:10^5^ (10^2^ cell/mouse spleen), acute infection can induce this frequency as high as10^7^, which encompasses almost 50% of all CD8+ T cells in LCMV-infected mice, or 44% of all CD8+ cells in human PBMC after EBV infection. In non-naïve recipients, activation of these cells with an epitope-based peptide can induce immunopathology with serious consequences.

LCMV-specific peptide epitopes injected into LCMV-immune recipient mice activated pre-existing virus-specific CD8+ T cells; the resultant TNF-dependent immunopathology killed the vaccinated mice within hours [115]. Injection of vaccinia virus-specific peptide B8R20-27 after vaccinia virus infection resulted in very rapid hypothermia, although all mice recovered. This concern is highly relevant for LASV infection in West Africa where most LASV infections are asymptomatic with seroprevalence as high as 25%-55% [36,67,98]. Furthermore, clinically healthy LASV-exposed individuals who may have lost their antibodies still had robust CMI responses to LASV recombinant proteins [60,61]. Based on epidemiological observations in West Africa, it is very likely that these individuals have LASV-specific have memory CD8+ T cells to effectively control LASV after the re-infection. Administration of an epitope-based vaccine to these individuals can strongly re-activate the pre-existing CD8+ T clone and induce TNF-dependent immunopathology with serious clinical consequences [115]. In summary, while injection of a peptide vaccine into naïve individuals might probably be safe, peptide- based vaccination may have severe pathological consequences in individuals recently infected with the virus or in immune individuals previously exposed (perhaps unknowingly/asymptomatically) to the pathogen. In addition, (i) currently there is no licensed peptide vaccine, the concept is unproven; (ii) since in West Africa the variability of the human HLA genes is very high, a large number of peptides would potentially have to be included in the vaccine, raising a concern regarding the feasibility of this approach.

### 4.3. Alphavirus Vector-Based Vaccines

Alphaviruses are small positive-sense RNA viruses with genome of ~ 11.5 kb encoding two open reading frames for 4 non-structural proteins (nsp1-4, the replicase cassette) and structural proteins (capsid, c; and glycoproteins, gp). The nsp1-4 proteins are translated from the genomic RNA and structural proteins are translated from the subgenomic 26S mRNA which are produced in 10-fold molar excess [116]. Alphavirus replicon particles, RP, are generated by deletion of structural genes from the replicon RNA and replacing them with heterologous gene(s) of interest. Co-transfection of cells with replicon RNA and helper genes provided *in trans* results in the packaging of the replicon RNA into alphavirus-like particles. The most developed alphavirus replicon vectors are based on Venezuelan equine encephalitis virus (VEEV), Sindbis (SINV), and Semliki Forest virus (SFV) genetic backbones (rev. [117]).

To some extent, alphavirus replicon technology provides a reasonable compromise, in terms of safety and immunogenicity, between “killed” vaccines and replication-competent platforms. Alphavirus RP are single-cycle, replication-defective vehicles (vectors). They are not able to spread beyond the initially infected cells, but can deliver and transduce the gene(s) of interest in target cells. Direct comparison of the immune responses induced by alphavirus-vectored vaccines and inactivated vaccines in mice showed clear advantage of aphavirus-vectored platform [118]. The alphavirus RP also offers attractive features in terms of safety, immunogenicity, and efficacy and currently numerous vaccine candidates are in pre-clinical and clinical development (rev. [117]).

One of the features of alphavirus RP is tropism to DCs. In contrast to “nucleic acid-free” VLP that have limited capacity to induce innate immunity, alphavirus RP induce robust innate immune responses. In recent study VEEV-based RP alone (without a transgene, “null” RP) were used as an adjuvant and dramatically improved the immunogenicity and protection efficacy of a licensed inactivated influenza vaccine in rhesus macaques [119]. Notably, the “null” RP induced mucosal responses even when the particles and immunogen were delivered via systemic routes [120,121]. It was also demonstrated that the alphavirus RP acted as a potent adjuvant to promote CD8+ T cells responses to co-delivered antigen [122], the feature which is especially attractive for LASV vaccine design.

VEEV-based replicon technology was successfully used to make mono- and bivalent vaccines expressing LASV NP, GPC and Ebola virus (EBOV) GP genes [96,123]. LASV genes were cloned downstream from the VEEV replicase and the 26S promoter in the transcription plasmid that contained the VEEV replicon cDNA. The LASV-replicons and two helper RNAs (c and gp) were prepared by *in vitro* transcription of the recombinant plasmids using T7 RNA polymerase and were used for co-transfection of BHK-21 cells. At 30 h post-transfection, the titers of the LASV-NP and LASV-GPC replicons in the medium of cotransfected cells were 10^7^ and 10^8^ IU/ml, respectively. The LASV RPs were negative for VEEV and expressed LASV NP and GPC. All strain 13 guinea pigs immunized with LASV-NP, LASV-GPC, LASV-NP plus LASV-GPC (5 animals/group) were protected against challenge with 160 LD_50_ of LASV (Jos). Low (at the level of detection), transient viremia was detected at day 7 after challenge in 3 animals vaccinated with LASV-GPC, and in one guinea pig in the LASV-NP group and in the LASV-GPC plus LASV-NP group [96]. Notably, immunization with RP expressing LASV NP and GPC genes induced robust antibody responses detected by IgG ELISA but not in plaque neutralization assay.

The VEEV-based replicon technology was also used to make a bivalent replicon for simultaneous expression of glycoprotein genes of LASV and EBOV. Vaccination of guinea pigs with dual-expression particles protected the animals against challenges with both viruses, LASV an EBOV [96].

**Figure 2:**
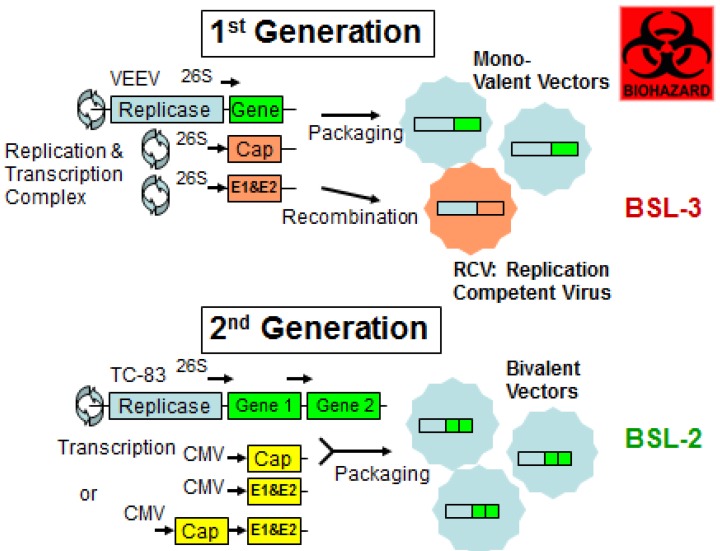
Advanced alphavirus-based vector technology. The 1st generation was based on Venezuelan equine encephalitis virus (VEEV) and required BSL-3 containment. Replication-competent virus (RCV) can be generated during a recombination event. In the 2nd generation, vectors use human vaccine VEEV TC-83 vaccine backbone and transcription of helper genes is controlled by non-related CMV (cytomegalovirus) promoter. Helper proteins (capsid and glycoproteins, E1&E2) can be made from two mono-cistronic CMV-plasmids or from one CMV-bicistronic plasmid (see text). Recombination events are minimized and BSL2 containment is required for production ([126]; Pushko & Lukashevich, unpublished.

The results summarized above indicate that replication-incompetent vectors that target antigen-presenting cells to deliver rationally-designed multivalent cross-protective arenaviral immunogens represent a feasible and attractive strategy. In the 1st generation of alphavirus-based replicon technology [123,124,125], replication-competent virus (RCV) can be generated by recombination of helper RNAs with replicon RNA (Figure 2). During packaging, recombination could potentially occur between replicating vector and helpers by template-switching mechanism, which could lead to regeneration of RCV, a live VEEV virus. Although helpers were split into c and gp parts (Figure 2) and attenuating mutations were introduced into helpers, the 1st generation of VEEV vectors required a BSL-3 containment for vaccine manufacturing [123].

To minimize the possibility of RCV regeneration and improve vaccine safety, a backbone of human TC-83 vaccine (IND No. 142) was used for making replication expression vectors and the packaging helper system (Figure 2) in the 2nd generation of this technology. The alphavirus 26S promoter for helper genes has been substituted with alphavirus-non-related CMV promoter and packaging helpers have been replaced with non-replicating constructs. These modifications will eliminate the possibility of

RCV generation. ). Additionally, the replicon expression vector has been re-designed to clone up to 5-kb-foreign insert and to express two (or more) foreign antigens. The multivalent feature of this system is certainly beneficial to optimize LASV vaccine formulation (e.g; simultaneous expression of GPC and NP genes); to address LASV genetic diversity (e.g; to express GPC from distantly related clades, I and IV); and to enhance immunogenicity of experimental vaccines (e.g; to express “wild-type” GPC for conventional antigen presentation and metabolic stable GPC for cross-priming CD8+ T cell responses). This technology is currently used to manufacture a bivalent cross-protective LASV vaccine for preclinical studies.

### 4.4. DNA Immunization

The authors of the first publication on LASV DNA vaccine [127] concluded that “DNA vaccine against LASV should encode as much of the proteome as possible, including both Lassa NP and GP”. In recent review, Grant-Klein et al. [128] noted that LASV DNA expressing GPC fully protected guinea pigs and cynomolgus macaques against LASV challenge. However, no results or details were provided. A LASV DNA vaccination approach was tested in LCMV murine challenge model based on the existence of common CD8+ T cell epitopes inducing cross-protective immunity. This model provides a quick and reliable test of a vaccine’s ability to induce CMI. It was shown that LCMV NP expresses the immunodominant CTL epitope (NP_118-126_) for H-2^d ^mice [129], and within the epitope, a minimal tetrapeptide GVYM is identical for LCMV, LASV, MOPV and plays an important role in CTL recognition [130]. Recombinant LASV NP protein expressed in vaccinia or in *Salmonella* protected 33% of mice from LCMV challenge, and the protection was associated with cross-reactive CTL and proliferative responses [131,132]. Similarly, a DNA vaccine expressing LASV NP showed partial (50%) cross-protection against LCMV and Pichinde virus (PICV) challenges in mice. DNA vaccination with a single epitope (LASV NP_118-126_) also partially protected mice from LCMV but not from PICV i.c. challenge [127]. Protection induced by the LASV NP_118-126 _minigen vaccine in this challenge model provided some rational for making multi-CD8+ CTL epitope constructs (“string of beads” vaccines [133]). However, these approaches share the negative consequences of the application of an epitope-based strategy in population with high prevalence of individuals previously exposed to LASV (see above) and therefore may not be applicable for LF vaccine.

Electroporation has recently emerged as a powerful tool to improve immunogenicity of DNA vaccines. In animal studies, electroporation enhanced efficacy of DNA vaccines by 10-1000-fold. However, phase I/II clinical trials showed the most common adverse effects are pain and bleeding at the injection sites. Electroporation is likely to be unsuitable for prophylactic vaccination, especially in children. However, this approach can be applicable for therapeutic cancer vaccines [134].

### 4.5. Rationally Designed Replication-Competent Vaccines

Replication-competent, “live-attenuated”, vaccines are among the most cost-effective and widely used public health interventions. The vaccines for smallpox, polio, and yellow fever dramatically reduced the incidence of these infectious diseases. However, successes in life science, biotechnology, and public health changed the emphasis on the effectiveness of vaccines to the safety of vaccines. This paradigm shift has led to the irony that the present regulatory standards would probably prevent the licensure of live-attenuated vaccines today. Currently, advances in molecular virology and rational design provide new opportunities for development of the next generation of these vaccines that optionally balance safety and effectiveness [135].

In most cases, LASV infection does not result in manifested disease, with only about 20% of infected people displaying the symptoms of the disease, which range from flu-like symptoms to fatal LF [2,9]. Long-term epidemiological observations in West Africa suggest that in immunocompetent adults, a single naturally “attenuated” infection can induce lengthy protection against fatal LF, and re-infection with heterologous strains will provide boosting effects [36,136]. While a high level of annual re-infection (up to 18%) is one of the characteristic features of LASV infection [5,98,136], a second clinical attack has never been documented [9].

There are several reasons to justify a replication-competent, “live” vaccine as an attractive approach to control LF: (i) CMI plays the major role in LF patient recovery and protection; (ii) a live vaccine provides the most effective natural pathway to process and present protective antigens to MHC molecules; (iii) epidemiological observations in LF endemic areas of West Africa provide evidence that a single (survived) exposure will induce long-term protection against disease [36]; (iv) a vaccine candidate formulated to contain both LASV NP and GP antigens will induce a broad cross-reactivity and a large pool of CD4+ memory T cells against all phylogenetic groups of LASV [60,61,137]; (v) a single-shot immunization approach is a crucial for population of remote rural areas of West Africa, which has a very limited medical infrastructure and where implementation of prime-boosting immunization is not practical.

The first vaccine for the prevention of viral hemorrhagic fevers caused by arenaviruses is Candid #1, a live attenuated vaccine. This vaccine is safe, highly immunogenic and efficacious. In 2007, this vaccine was included into the Argentine National Immunization Plan and significantly reduced morbidity and mortality caused by JUNV infection [138]. Currently there are four replication-competent LASV vaccine candidates based on vaccinia virus [94,139,140], vesicular stomatitis virus (VSV) [141,142], MOPV [48,137,143], and yellow fever 17D [144,145] vectors. All these vaccine candidates have been tested in NHP and will be described in more detail.

#### 4.5.1 Recombinant vaccinia virus expressing LASV antigens

The first experimental vaccine successfully tested in animals was a recombinant vaccinia virus (Lister) expressing NP of LASV strain GA391 isolated from a patient in northern Nigeria [94]. The NP gene was placed in the transfer vector pGS20 and subsequently inserted into the thymidine kinase gene of vaccinia under control of the 7.5K promoter. Six outbred (Dunkin-Hartley) guinea pigs inoculated with of 10^7.5 ^PFU (s.c.) of recombinant vaccinia were fully protected against fatal challenge with 10^4^ TCID_50_ (tissue culture infectious doses) of LASV GA391. Protected animals had no clinical signs of the disease and viremia was not detected.

Cloning of both LASV (Josiah) major antigens, NP and GPC, into the vaccinia vector (NYBH) gave similar results as described above; 94% of outbred guinea pigs vaccinated with LASV-NP (V-LSN) and 79% vaccinated with LASV-GPC (V-LSG) were protected against LASV (Jos) challenge [146]. Protective efficacy of vaccinia recombinant V-LSG was confirmed in rhesus monkeys (*Macaca mulatta*) [147]. Four animals were vaccinated with 10^9^ PFU of V-LSG and two rhesus monkeys received MOPV (10^4^ PFU, a positive vaccination control). Vaccinated animals were challenged on day 37 with LASV (Jos, 10^4^ PFU), and survived the challenge. Low transient viremia (10^2^-10^3^ PFU/ml) was detected in all survived animals on day 7-9 after challenge. In spite of complete recovery, V-LSG-vaccinated animals developed a mild fever, some chemical and hematological abnormalities (depressed platelet function, lymphopenia and neutrophilia) which were not observed in MOPV-vaccinated/LASV-challenged animals. After vaccination, only two V-LSG animals developed antibodies to LASV GP, and no serum from any animals after vaccination had any neutralizing activity, which again emphasizes the importance of CMI over humoral immunity.

A broader study in 44 NHPs (28 rhesus and 16 cynomolgous) that summarized vaccination/challenge experiments with recombinant vaccinia that expressed different parts of GPC, NP, provided more detail on the efficacy of vaccinia-based recombinant vaccines and contribution of individual antigens to protection [140]. These results showed that both glycoproteins, GP1 and GP2, are required for protection, and that the full length GPC is necessary and sufficient to protect animals against challenge.

In contrast to results in guinea pigs, 12 out of 15 animals vaccinated with NP died (20% survival), although this vaccination developed a strong antibody response before challenge. Animals vaccinated with GPC had low antibody titers before challenge, but 17 from 19 vaccinated were fully protected (88%). The highest level of protection (90%) was observed when animals received all proteins encoded by the LASV S RNA (V-LSG/N or V-LSG + V-LSN). In these two groups, the lowest levels of transient viremia, comparable with levels in MOPV-vaccinated group (a vaccination control), were detected. No neutralizing antibodies were detected in any animal groups before or after challenge. This study confirmed that antibody plays a minor, if any, role in virus clearance and protection. It was also demonstrated that challenge dose, as well as the interval between vaccination and challenge can affect outcome of the experiments [9,36,140]. This study also highlighted a controversial role of LASV NP in protection and the relative importance of a reliable and validated animal model for evaluation of LASV immunogenicity and efficacy (see below).

#### 4.5.2. Replication-competent VSV vectors expressing LASV glycoproteins

Initially, recombinant vaccinia virus expressing LASV genes provided promising results and helpful information for LASV vaccine development. However, this vaccine platform is no longer considered as acceptable because of potential safety issues associated with the immunosuppressive phenotype of the vector. The prevalence of immune-suppressed individuals is especially high in Sub-Sahara Africa. Nigeria has the highest number of clinical cases of LF and it also ranks as the second worst HIV-1-affected country in the world (after South Africa). In our preliminary studies, we found that LASV sero-prevalence was 3-times higher in HIV-positive individuals than in the HIV-negative group (Abimiku & Lukashevich, unpublished) indicating that both infections co-exist in Nigeria and can target the same population.

Attenuated recombinant VSV (rVSV) vector was proposed as a reasonable alternative to a vaccinia virus platform. The VSV is a negative-stranded, non-segmented RNA virus belongs to the *Rabdoviridae, *genus *Vesiculovirus*. The virus infects a wide range of animals, replicates very well in different types of cells, and induces a strong cytopathic effect. The genomic RNA encodes five major proteins, glycoprotein (G), matrix protein (M), nucleoprotein (N), large protein (L) and phosphoprotein (P). There is a powerful molecular biology tool to genetically manipulate VSV; the rVSV lacking its own G gene has been widely used to produce pseudotypes with envelope proteins of heterogeneous viruses (rev. [148]). The G is the major pathogenic factor and replacement of VSV G with a heterogeneous type I transmembrane glycoprotein results in generation of attenuated rVSV, incorporating the foreign glycoprotein on the bullet-like VSV surface. Based on this feature and the ability to tolerate foreign transcription units, rVSV was successfully used as a promising vaccine vector for varieties of pathogens; among them, rVSV expressing EBOV and MARV glycoproteins, were extensively tested in NHPs with encouraging results (rev. [149,150]).

The filovirus GP cloning strategy applied for LASV GPC resulted in generation of recombinant VSV∆G/LASVGP particles with bullet-shaped nucleocapsid morphology [141]. For both recombinant viruses, (VSV∆G/LASVGP and VSV∆G/ZEBOVGP), growth kinetics in Vero cells at high m.o.i. were attenuated, reaching maximum titers 24 and 36 h post-infection, respectively. Western blot analysis detected the LASV glycoprotein precursor, GPC, in infected cells and proper cleavage into GP1 (44 kDa) and GP2 (36 kDa). Immunoelectron microscopy using monoclonal antibodies against LASV GP1 and GP2 confirmed expression of LASV glycoproteins on surface of recombinant virus particles [151]. As expected, replacement of VSV gene with LASV GP resulted in cell tropism changes. The wild-type VSV infects and replicates in human T-cell leukemia cells (Jurkat), but these cells are not susceptible for LASV and EBOV. Recombinant VSV∆G/LASVGP and VSV∆G/ZEBOVGP failed to replicate in these cells as well [141].

Recombinant VSV∆G/LASVGP (2 x 10^7^ PFU) was inoculated (i.m.) into four cynomolgus macaques [151]. Immunized animals did not display any clinical symptoms. Twenty-eight days after immunization, vaccinated and control monkeys were challenged with 10^4 ^PFU of LASV (Jos). The control animals developed a severe LF and were euthanized on days 11 and 12. All vaccinated animals were fully protected and survived the LASV challenge without clinical manifestations. Hematology and blood chemistry analysis revealed slight perturbations in platelet numbers and in plasma ALT levels in vaccinated animals after LASV challenge. Although viremia was not detectable after immunization, LASV was transiently detected in all vaccinated animals at low (10^2^ PFU/ml) to moderate (10^4^ PFU/ml) levels, indicating that immunization did not induce sterilizing immunity. All 4 vaccinated macaques developed moderate- to high levels of IgG ELISA antibodies and low levels of neutralizing antibodies before challenge. CMI responses were detected by intracellular staining of CD4+ and CD8+ lymphocytes for secretion of IFN-γ and TNF-α. Secretion of these cytokines was found in CD8+ cells only in one vaccinated monkey before challenge. LASV challenge markedly increased antibody responses (ELISA, neutralization) in all animals. Surprisingly, LASV challenge stimulated CMI responses only in 3 of 4 protected animals, indicating that protection at least in one animal was not associated with activation of CD8+ or CD4+ T cells. Although mechanism of protection induced by rVSV∆G/LASVGP is not still clear and some safety concerns still need to be addressed, this vaccine candidate is well positioned to further development. Recent progress with filovirus vaccines based on the same technology should also provide a helpful guidance.

#### 4.5.3. Reassortant vaccine platform, MOP/LAS (clone ML29)

The bi-segmented feature of the arenavirus RNA genome indicates that these viruses can produce reassortants during co-replication *in vitro*. Nevertheless, natural reassortance between arenaviruses has not been reported; although there is an evidence for natural genetic recombination within the S RNA segment of the NW arenaviruses [152]. Intertypic reassortants within LCMV [153,154,155] and PICV [154,156] species and between two different species (LASV and MOPV) [48,49], were generated *in vitro*; these reassortant viruses were successfully used for coding assignment and for understanding the molecular basis of virus pathogenicity. Reassortant analysis indicates that the L RNA is important for high levels of virus replication *in vivo* and it is associated with acute disease in experimental animals [155,156].

The MOP/LAS reassortant, clone ML29 [12,157], was designed to keep the attenuated profile of MOPV, but to induce a robust cross-protective CMI responses against genetically-distant LASV isolates. Clone ML29 was selected from a library of MOPV/LASV reassortants and encodes the major antigens (NP and GPC) of LASV, as well as the L protein (RdRp) and Z protein of MOPV [48]. Since clone ML29 has 70% of MOPV genome, it has the same risk classification as the vector virus, MOPV. MOPV is considered risk group 2 by the EU biosafety regulation and risk group 3 by the CDC. 

While co-infection of Vero cells with MOPV and LASV resulted in production of stable reassortants, our attempts to isolate viable reassortants after co-infection of cells with LASV and LCMV failed. Recent experiments using minireplicon systems developed for these viruses explained these results. The minigenoms (MGs), NPs, and L proteins of LASV and MOPV were exchanged and interacted homologously and heterologously without loss of function. L proteins of LASV and MOPV were also active in interaction with LCMV NP. However, the LCMV L protein required only homologous NP for its activity [47]. Transcriptional replicon activity of MGs was measured via expression of the Ren-Luc reporter. When activity of 3 minireplicons was compared, the 10-fold-reduced activity of the MOPV system compared to the LASV and LCMV systems was found [47]. Interestingly, the substitution of the MOPV 3′-UTR by that of LASV increased the expression of Ren-Luc from the MG suggesting that translation is initiated less efficiently at the MOPV 3′-UTR than at the LASV 3′-UTR. It is intriguing to find evidence how this “attenuated” initiation translation is associated with attenuated phenotype of MOPV.

Eighteen mutations distinguish the ML29 genome from the parental arenaviruses, MOPV (L RNA) and LASV (S RNA) and likely additionally contributed to the attenuated phenotype [137,157] (Table 1). The ML29 genome was stable during 12 passages in tissue cultures and all these mutations were preserved as it was shown by deep sequencing of ML29 isolates. Among 18 mutations, noncoding transversions A6C, U8G and A7264C, U7266G were located within the 3’ and 5’ termini of the L RNA (derived from MOPV) and one mutation, G3328A was identified within the long 3’ non-coding region of the S RNA (derived from LASV) of ML29. A recently developed LASV reverse genetic system was applied to investigate the contributions of noncoding regions of the LASV S RNA on growth kinetics *in vitro* and virulence in mice [158]. This approach showed that deletions of large portions of these noncoding sequences resulted in attenuated LASV replication *in vitro* and *in vivo*.

The terminal 19 nucleotides of each RNA segment are highly conservative among arenaviruses and represent the promoter directing the virus genes expression and genome replication [159,160]. These sequences are mostly complementary to each other, since perfect complementarity within the terminal UTRs is required for efficient RNA replication [161]. Mutational analysis of LASV promoter in the S RNA MG system showed that this promoter coordinates transcription and replication. It contains two regions, a sequence-specific region (residues in positions 1-12) and a variable complementarity region (positions 13-19) [159]. Mutations distinguishing the parental MOPV L RNA sequence from the analogous terminal sequences of the ML29 L RNA were located at positions 6 and 8. Mutations in these positions were tolerated, consistent with some natural variability among arenaviruses. Notably, these mutations suggest that the predicted pan-handle secondary structure of the ML29 L promoter is more stable than those in the parental viruses, LASV and MOPV [12,157].

Mutations in the coding region of the L RNA resulted in 3 non-conservative amino acid substitutions at positions 851(Tyr→Asn), 1233 (Arg→Gly), and 2136 (Asp→Asn). Only the R1233G

**Table 1:** Mutations in genomic segments and proteins of attenuated Mopeia (MOP)/LAS reassortant, clone ML29 [137,157].

Mutations/Substitutions	Location	Putative function	Possible effects (References)
A6C, U8C	L RNA, 5’, 3’ NCR	Form panhandle	Enhance promoter stability [12]
A7264C			
A7264C			
G3328A	S RNA, 3’ NCR	Unknown	Deletions in the 3’NCR attenuate replication [158]
Y851N	L protein, N-term.	Cap-snatching	Attenuated transcription/ replication [162,163]?
N173S	NP, central domain	Interaction with RNA	Unknown
A485D	NP, C-terminal	Exonuclease	IFN modulation [164,165]
K272E	GP2, N-terminal	Fusion	Affect fusion/post-fusion events?

mutation was located within the putative RdRp module and mapped between motif A, Asp-X(2)-Lys-Trp, and motif B, Gly-X(5)-Ser. These motifs represent stretches of highly conserved sequences that are linked by less conserved variable regions [162]. The Y851N substitution was located between N-terminal and an RdRp domain located in the central part of the molecule. A large scale mutagenesis approach found that an N-terminal region of LASV L protein plays a crucial role in transcription but not in replication of the LASV genome. The so called “L1 domain” (from position 1 to 300) can be a part of an endonuclease involved in putative a cap-snatching mechanism required for mRNA synthesis [163]. The N-terminal domain of MOPV L protein seems to be involved in the similar activity. The functional role of a C-terminal region where a D2136N substitution was found is currently unknown.

The S RNA of ML29 was derived from LASV and differs in sequence by six mutations. These mutations resulted in three amino acid substitutions, two of them were located in NP and the 3rd mutation was identified in N-terminal domain of GP2. The homologous NP mutation, N173S, was mapped in the central region of the protein, and a non-conservative substitution, A485D was located in the C-terminal region of NP which was critical for immunosuppressive function [166,167,168]. Crystal structure of LASV NP showed that this mutation is located between two aspartic acid residues at positions 466 and D533 which involve in formation of active site of the putative LASV NP exonuclease associated with the NP ability to suppress translocation of IRF-3 and to block IFN activation [164,165,169]. This site contains two critical residues, D389 and G392, and recombinant LASV mutants carrying mutations at these residues were severely altered in their ability to suppress IFN in DCs and macrophages.

**Figure 3:**
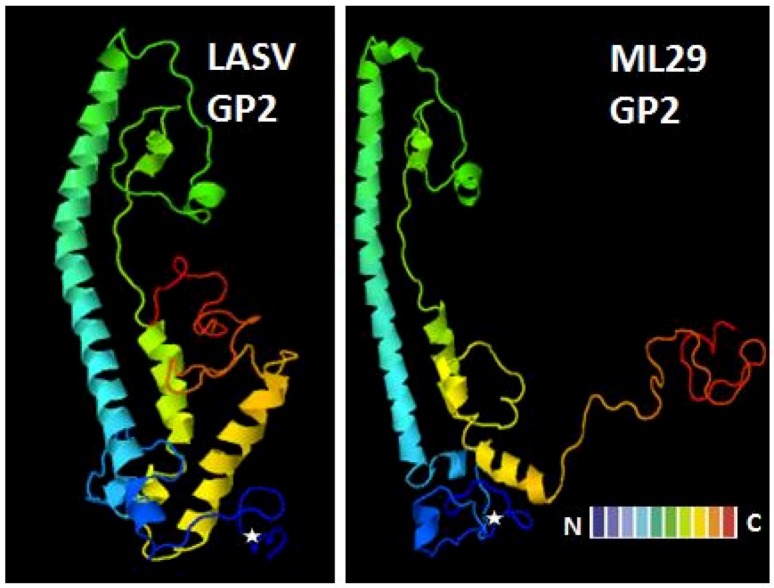
The predicted structure of GP2 glycoprotein of ML29. The non-conservative K272E substitution (marked by star) located between two fusion domains at N-terminus introduced two negatively charged groups in positions 272-273 (Asp-Glu) resulted in refolding and exposure of transmembrane and cytoplasmic domains. Protein structure prediction was performed using the Phyre server (http://www.sbg.bio.ic.ac.uk/phyre2/html/page.cgi?id=index) [173].

Both viruses, LASV and ML29, have identical surface-exposed GP1 responsible for interaction with cell receptors; while ML29 GP2 is different from LASV GP2 by the K272E substitution located between two fusion domains. The transmembrane GP2 is the class I fusion protein. We for the first time demonstrated the fusion activity of an artificial peptide derived from highly conserved hydrophobic region at amino acid positions 276-298 [170]. Later, Klewitz et al. [171] confirmed these results and found that the N-terminal stretch, residues 260-266, located downstream of the GPC cleavage site is also involved in the fusion activity. Notably, all LASV strains analyzed so far have mutations between two domains with fusion activity, at positions 272-274 (K-D-T for LASV Josiah GP2). In ML29 GP2 Lys to Glu substitution introduces two negatively charged groups in positions 272-273 (Asp-Glu) distinguishing the MOP/LAS reassortant from other LASV strains analyzed so far [137,172]. These changes significantly affect the predicted GP2 structure (Figure 3) by refolding and exposure of C-terminal part of the GP2 with transmembrane domain and a cytoplasmic tail. This rearrangement of GP2 transmembrane domain can potentially affect fusion and/or post-fusion events during ML29 entry. Interestingly, the F427I substitution found in GP2 transmembrane domain

of JUNV Candid #1 vaccine seems to be involved in the destabilization of the GP metastable conformation and probably contributes to attenuation [174]. At least virus entry/fusion directed by Candid #1 GP2 was significantly less affected by NH_4_Cl (to block the endosomal acidification) than the entry mediated by wild type GP2 [174]. Key role of the F427I substitution in attenuation of Candid #1 was recently confirmed by reverse genetics [175] and this mutation was proposed to be a molecular signature of JUNV attenuation [174]. Interestingly, we [12] and others [176] also observed different effects of ammonium chloride treatment on LASV and MOPV replication kinetics in cell cultures.

The immunogenicity and efficacy of ML29 vaccine candidate was tested in all available animal models to comply with the FDA Animal Rule [177] and included mice, guinea pigs, and NHP (rhesus macaques and common marmosets). Although mice do not accurately model human LF disease, they can provide an economical assay for vaccine potency in terms of capacity to elicit CMI and recentstudies demonstrated correlation between LASV-specific CD8+ cytotoxicity *in vivo* and ML29-induced protection [178]. The ML29 immunization with 150 PFU and higher produced immune splenocytes that completely protected animals against a fatal LASV challenge [137]. Protective T lymphocytes were detected in spleen as early as 2 days after vaccination. The cells collected on day 5 and 7 protected 60 and 100% of mice, respectively. Protection was dose-dependent and required 3 x 10^7^splenocytes to protect all recipient mice against fatal LASV challenge. LASV infection of CBA mice resulted in rapid accumulation of the virus in tissues and the death of animals at day 6-7 after i.c. inoculation [179]. Injection of the ML29-immune splenocytes effectively eliminated the virus from tissues of challenged animals. At day 3 after immune lymphocyte transfer, the virus was undetectable in spleen and other tested tissues (brain, liver, kidneys) of recipient mice. The effective virus clearance seems to be the main mechanism of protection of mice treated with ML29-specific T lymphocytes. 

A single injection of ML29 provided full protection of strain 13 guinea pigs against more than 3,300 LD_50_, animals did no express any clinical or biochemical abnormalities during the observation period (70 days). Meanwhile, in MOPV-vaccinated animals, a transient elevation of AST and alkaline phosphatase in plasma was observed at week 3 after challenge, suggesting liver injury [137]. Histology studies did not reveal lesions in tissues of ML29-vaccinated animals that were challenged with LASV.

Genetic diversity of LASV has been a great challenge for vaccine development. The highest sequence differences (based on partial NP sequences) were found between lineages II (803213/NIG/74/H) and IV (JOS/SL/76/H), 11.0-14.4%. To determine whether the ML29 vaccine can protect strain 13 guinea pigs from distantly related LASV, vaccinated animals were challenged with LASV-JOS or with LASV-NIG. The S RNA of ML29 derived from LAS-JOS strain [137,157] and challenge with this virus was considered as a homologous vaccination-challenge protocol. 

As seen in Table 2, all ML29-vaccinated animals were completely protected against challenge with two LASV strains, Josiah/SL (group 7) and 803213/NIG (group 8), and had no clinical manifestations of the disease. However, ML29-vaccinated animals were not protected from challenge with LCMV-WE, although vaccinated animals lived longer than animals in the control group, 16-21 days vs 13-14 days (group 9). These observations indicate that ML29 vaccination can induce some levels of cross-protection against LCMV-WE.

**Table 2:** Results of vaccination and challenge experiments in guinea pigs [180].

Animal Group		Challenged virus	Dose, PFU	Vacc/chal interval, days ^a^	No. survived/No. infected	Survival, %	Day of death
No vaccination							
1		LASV-Jo	10e+1	na ^b^	0/4	0	15-17
2		LASV-Jo	10e+3	na	0/5	0	15-16
3		LASV-803213	10e+3	na	0/5	0	13-15
4		LCMV-WE	10e+3	na	0/5	0	13-14
The ML29 conventional vaccination (challenge on day 30)							
5	10e+2	no challenge	na	na	6/6	100	na
6	10e+6	no challenge	na	na	6/6	100	na
7	10e+3	LASV-Jo	10e+3	30	6/6	100	na
8	10e+3	LASV-803213	10e+3	30	5/5	100	na
9	10e+3	LCMV-WE	10e+3	30	0/6	0	16-21
The simultaneous vaccination/challenge experiments (challenge on day 0 and 2)							
10a.	10e+6	LASV-Jo	10e+1	0	5/5	100	na
10b.	10e+6	LASV-Jo	10e+1	2	3/5	60	10^c^,15
11a.	10e+6	LASV-Jo	10e+3	0	4/4	100	na
11b.	10e+6	LASV-Jo	10e+3	2	4/5	80	10 ^c^
12a.	10e+2	LASV-Jo	10e+3	0	3/4	75	14
12b.	10e+2	LASV-Jo	10e+3	2	3/4	75	16
13	10e+6	LASV-803213	10e+3	0	3/5	60	12,17
14	10e+6	LCMV-WE	10e+3	0	0/5	0	14-16

^a ^Animals were s. c. vaccinated with the ML29 reassortant (day 0) and challenged simultaneously on day 0, 2, 30 after vaccination. Death or survival past 21 days was set up as an endpoint. Amino acid difference between LASV-Jo and LASV-803213 is the highest within LASV genetic lineages I-IV.

^b ^Non applicable; ^c ^Non-LASV-specific death (inappropriate anesthesia).

Several examples of natural reassortment between genetically-related bunyaviruses raised the concern that vaccination with an attenuated reassortant vaccine in endemic areas will affect the natural pattern of the disease and enhance pathogenicity. To address this concern challenge and vaccine application was performed simultaneously (day 0). All animals vaccinated with 1 x 10^6^ PFU of ML29 survived after simultaneous challenge with low (group 10a) or high (group 11a) doses of LASV-Josiah. Importantly, the vaccination also protected 60% animals from challenge with high dose of the distantly-related strain, LASV-803213/NIG (group 13). Vaccination with low dose, 1 x 10^2^ PFU of ML-29, protected 75% animals against challenge with high dose of LASV-Josiah. Vaccination two days before challenge was also effective protecting more than 60-80% of the guinea pigs against death.

In ML29-vaccinated animals challenged on day 30 with LASV-Jos or with distantly-related Nigerian strain viremia was not detectable by conventional plaque assay, co-cultivation on Vero cells, or by qRT-PCR at all tested time-points, on day 4, 8, 12, 21 after challenge. Therefore, this demonstrates that those animals challenged 30 days after vaccination were not only completely protected against disease but also against LASV infection suggesting that vaccination with ML29 induces sterilizing immunity. In partially protected animals clinical parameters were not as severe as in non-vaccinated animals and partial recovery on day 21 [180]. Non-neutralizing IgG ELISA antibodies against LASV, predominantly anti-NP, were detectable at the end of the 1st week of vaccination and peaked shortly after challenge. These antibodies apparently do play major role in the recovery and protection [180].

**Figure 4:**
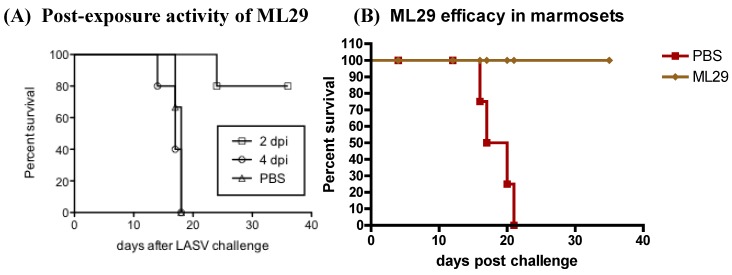
(A), Survival rate of strain 13 guinea pigs (4 animals per group) fatally infected with LASV (Jos, 1000 PFU, s.c.) and treated on day 2 and 4 after challenge with ML29 (1000 PFU, s.c.); (B), ML29 preventive efficacy in common marmosets. Animals (6 animals per group) were vaccinated with ML29 and challenged on day 30 with LASV (Jos) [143].

Protection of animals after simultaneous vaccination-challenge justified a post-exposure treatment of LASV-exposed animals. As seen in Figure 4, 80% guinea pigs were protected against fatal LF when animals were treated with ML29 two days (48 hours) after LASV challenge. This treatment resulted in clinical (temperature and weight) and biochemical (albumin, AST/ALT) improvements and recovery.The similar experiments are under way to test efficacy of ML29 post-exposure treatment of rhesus macaques fatally infected with LASV (R. Carrion, personal communication). 

Mechanisms of protection in postexposure treatment are not clear. Currently at least two hypotheses are under evaluation: (i) the ML29 L RNA has a more stable promoter and higher affinity to RdRp and effectively outcompete the LASV during co-replication in target cells (DCs, macrophages); (ii) the ML29 treatment will rescue fatally infected animals by activation of ML29-specifi innate immune responses overcoming LASV infection.

In rhesus macaques, a single injection of 1,000 PFU (s.c.) of ML29 resulted in induction of primary virus-specific T cells capable of secreting IFN-γ in response to homologous and heterologous antigen stimulation [137]. These cells can be detectable in peripheral blood and in spleen on day 7 after immunization. On day 28 after vaccination, among the ML29-specific cells, up to 13% of T cells cross-reacted in response to closely related viruses, LCMV-WE or MOPV [137]. ML29 immunization resulted in antibody responses detected in ELISA at week 2 after immunization and with an endpoint dilution titer of 1:62,500 on day 28. Neutralization antibodies were undetectable (<1:20) at this time-point.

**Table 3:** Viremia in marmosets vaccinated with ML29 and challenged on day 30 with LASV ^a^ [143].

Animal No	Time, days after challenge:					
	0	5	15	17-19	20	35
Non-vaccinated						
CJ25391	<1.0	2.95	4.32	nd	na	na
CJ26255	<1.0	+	4.45	5.54	na	na
CJ26494	<1.0	2.11	5.38	nd	na	na
CJ26950	<1.0	+	4.81	6.78	na	na
ML29-vaccinated						
CJ26493	<1.0	2.67	<1.0/-	<1.0/-	<1.0/-	<1.0/-
CJ27008	<1.0	<1.0/-	<1.0/ +	<1.0/-	<1.0/-	<1.0/-
CJ26190	<1.0	<1.0	<1.0/-	<1.0/-	<1.0/-	<1.0/-
CJ26946	<1.0	<1.0/+	<1.0/-	<1.0/-	<1.0/-	<1.0/-
CJ26256	<1.0	<1.0/-	<1.0/-	<1.0/-	<1.0/-	<1.0/-
CJ26249	<1.0	<1.0/-	<1.0/-	<1.0/-	<1.0/-	<1.0/-

^a ^Viremia in blood was measured by plaque titration and expressed as log_10_ PFU/ml. Average from two plaque titrations is presented. Viral load is expressed as log_10_ PFU/ml; <1.0, lower than detection limit; +, sample was positive in co-cultivation assay; <1.0/+, negative in direct plaque assay, but positive in co-cultivation assay; <1.0/-, negative in plaque assay and in co-cultivation assay

A shortage and high cost of rhesus macaques complicated efficacy trials, especially challenge experiments in a BSL-4 environment. To overcome this problem we recently developed a small non-human primate model of LF in the common marmoset, *Callitrichid jacchus* [84]. Immunization of marmosets with ML29 increased populations of CD14+ cells and CD3+ T lymphocytes in circulating blood. We also found recruitment of CD3+ T cells and over-expression of HLA-DR, P, Q in target tissues [181]. Taken together these data indicate that ML29 immunization resulted in antigen stimulation. In contrast, LASV infection in marmosets was associated with lymphoid depletion, marked reduction in CD3+, CD20+ cells, and down-regulation of class II MHC antigens [84].

A single s.c. inoculation of ML29 vaccine (1000 PFU) induced specific cell-mediated T cell responses assayed in IFN-γ or TNF-α ELISPOT and these responses seem to be responsible for full protection animals against LASV challenge (Figure 4B). No vaccinated animals had any clinical manifestations and all survived the observation period lasting 35 days after challenge. In vaccinated and challenged animals blood chemistry and hematology data did not reveal differences from pre-challenge values (not shown). In contrast, challenge controls showed reduction of platelet numbers, elevated liver enzymes, and decreased levels of albumin in plasma as was previously described [84].

Plasma samples collected after challenge were tested by plaque assay and by co-cultivation on Vero cells. As seen in Table 3, in all non-vaccinated marmosets LASV was detectable on day 5 after challenge and the viremia was more than 10^5^ PFU/ml shortly before or on the day of necropsy. Only in one ML29-vaccinated marmoset was LASV detected by plaque assay on day 5. Two other LASV-positive animals had low transient viremia detectable by Vero co-cultivation. After day 15, no animals had LASV in plasma as judged by biological assays or by nested RT/PCR. In tissues of non-vaccinated animals LASV load was high and varied from 10^3.3^ to 10^7.6^ PFU/g. All tested tissues of ML29-vaccinated animals that were challenged with LASV had no detectable infectious LASV [137].

In all tested animal models, including non-human primates [137,143], replication of ML29 was deeply attenuated, did not induce viremia, or any biochemical/clinical abnormalities. Only immunization at very high doses (10^6^ PFU, 1,000-fold higher then protective dose) resulted in low transient viremia in one of six tested monkeys [143]. Recent studies demonstrated that rhesus macaques infected with Simian Immunodeficiency Virus (SIV) and uninfected monkeys responded similarly to ML29 vaccination, and that none developed signs of arenavirus disease or persistence. Furthermore, 5 of 5 SIV-infected rhesus macaques vaccinated with ML29 animals given a heterologous challenge with a lethal dose of LCMV-WE survived without developing disease signs [182]. It indicates that ML29 is safe and immunogenic in SIV-infected rhesus macaques.

In a surrogate model of LF in rhesus macaques inoculated with viscerotropic WE strain of LCMV [72] early transcriptional changes in blood, mostly in genes involved in complement, IFN, and pro-inflammatory pathways, can discriminate virulent vs. non-virulent infection [183]. We applied the similar approach and showed that gene expression patterns in ML29-exposed human PBMC and control, mock-exposed cells, clustered together confirming safety profile of the ML29 in naïve primates [137,143].

In summary, the ML29 is a promising LASV vaccine candidate that: (i) elicits a strong cross-reactive CMI before challenge; (ii) completely protects guinea pigs and marmosets from LF and this protection is highly suggestive of sterilizing immunity; (iii) provides protection against heterologous LASV strains; (iv) protects fatally infected guinea pigs by treatment at 48 h following infection; and (v) safe and immunogenic in NHP with simian AIDS. Still, risk group classification of MOPV (“guilt by association” [9]) and, consequently, ML29 status as a risk group 3 pathogen, is the major obstacle for further development of this vaccine.

#### 4.5.4. Recombinant Yellow Fever 17D expressing LASV antigens.

There are several arguments to consider Yellow Fever 17D (YF17D) as a vaccine vector for expression of LASV antigens. The YF17D is one of the most efficacious and safe vaccines ever developed with a highly favorable benefit-risk profile and excellent cost-effectiveness. Since 1937 more than 540 million doses have been delivered world-wide and no reversion to wild-type YF virus has been reported. Rare observed adverse effects seem to be associated with host factors and are not due to virus reversion [184,185,186]. However, during the past 20-25 years the absence of effective public health policy, social-ecological factors and vaccine shortage resulted in a resurgence of YF (~ 200,000 cases and 30,000 deaths annually) in South America and Africa. Thirty-two African countries with a total population of 610 million people including more than 219 million urbanites are now considered at risk for YF. The vast majority of cases and deaths take place in sub-Saharan Africa [187], where YF endemic areas are overlapping with LF risk zones [3]. Fisher-Hoch & McCormick, well-recognizable leaders in LASV research, noted that “For the long term, a yellow fever/Lassa fever chimera vaccine for use in EPI in West Africa is a very attractive solution” [188].

Based on outstanding YF17D safety records and recent success in molecular biology of flaviviruses, the genetic backbone of YF17D has been used for construction of chimeric YF17D-based viruses expressing prM and E proteins of closely-related flaviviruses, Japaneses encephalitis (JE), Dengue, and West Nile virus. ChimeriVax™-based vaccines against these flaviviruses are currently undergoing Phase II-III clinical testing (reviewed by [189]). Insertion of short immunogenic peptides into E surface glycoprotein protein did not compromise the morphological structure and attenuated phenotype of YF17D. Recombinant YF17D-based vectors have also been used in the development of promising new vaccines carrying immunogenic epitopes of flavivirus-unrelated pathogens (influenza, malaria, oncogenes) [190,191,192,193,194].

ChimeriVax™ technology is not applicable for cloning large flavivirus-unrelated genes. To generate recombinant YF17D viruses expressing LASV genes we developed technology to clone and express LASV GPC between YF17D E and NS1 genes [144]. In brief, a cDNA fragment encompassing the complete sequences encoding LASV GPC, but lacking the 58 amino acids of the GPC signal sequence to prevent interference with YF polyprotein processing, was fused in frame between the YFV E and NS1 genes. The construct was designed so that LASV GPC protein would be released from the YF17D polyprotein by host signalase. In this construct, the C-terminal 23 hydrophobic amino acids of the YF17D E gene were duplicated downstream of the LASV GPC gene to serve as a signal sequence to ensure insertion of the YFV NS1 protein into the endoplasmic reticulum (ER). In vitro-made RNA from the recombinant clone and from the parental YF17D clone was transfected into BHK-21J cells by electroporation and the recombinant virus was collected from culture medium.

The YF17D/LASV∆GPC recombinant virus was replication-competent, deeply attenuated, induced immune responses against both pathogens, YFV and LASV, and protected 80% of guinea pigs against fatal LF in proof-of-concept challenge experiments [144,195]. In spite of these encouraging results, we [144] and others [196] faced instability problems when large inserts were cloned at the E-NS1 site. It has been suggested that duplication of flavivirus sequences flanking an inserted gene contributed to the genetic instability of the virus. Two approaches were applied to address this problem: (i) insertion of modified fusion sequences between the C-terminal part of foreign inserts and NS1 gene to prevent possible recombination events resulting in deletion of the foreign gene; and (ii) reduction of insert size. We have shown that modification of fusion sequences improved stability but did not preserve the large ∆GPC insert from the deletion during 10 passages in tissue culture. When the size of the insert was reduced almost 2-fold, GP1 and GP2 sub-units of LASV GPC were successfully cloned at the E-NS1 site and these inserts were stable during 10 passages. The size of the LASV GP1 is 200 aa and the size of GP2 is 231 aa residues. In accord with our results, a similar sized insert, 224 aa residues, from SIVmac239 Gag was cloned between the genes encoding the viral proteins E and NS1. The resulting recombinant virus, the YF17D/SIVGag_45-269_, was stable in vivo and able to induce SIV-specific CD8+ T cell responses in rhesus macaques [197].

The size limitation of foreign gene inserts in YF17D vector seems not to be unique for the E-NS1 site. Recently recombinant YFV17D vaccine was engineered by cloning CSP (aa 57-344) from P. yoelii using a novel insertion site, the capsid gene. The insert was cloned in frame between the highly conserved cyclization sequences (nt 1-75) and FDMV 2A and Ubi genes which were inserted to ensure appropriate cleavage [198]. Notably, the CSP insert was intact up to 6 passages in tissue culture and became undetectable by RT/PCR by passage 7. We have used a similar strategy with limited success in attempts to clone and express overlapping fragments of LASV NP resembling in some ways cytoplasmic YFV core protein (P. Bredenbeek et al; unpublished).

Taken together these results indicate that a monocistronic YF17D genome encoding a relatively large flavivirus-nonrelated gene inserts at the E-NS1 site or in the C gene can produce viable recombinant viruses in cell cultures. However, genetic stability of recombinant viruses is the major problem.. We have shown here that cloning of inserts of moderate size (200-230 aa residues) between the genes encoding the viral proteins E and NS1 resulted in genetically stable recombinant viruses during 10 passages in tissue culture. The YF17D genome itself is remarkably stable [199]. Ten passages of recombinant YFV17D-based viruses will be sufficient to manufacture non-GMP lots for toxicology and pre-clinical studies and for making Phase I lots during vaccine development [189].

Insertion of foreign genes into the YFV17D genome resulted in additional attenuation of chimeric or recombinant YF17D-based viruses. In plasma and tissue of vaccinated guinea pigs [144] and marmosets (I. S. Lukashevich, unpublished) YF17D/LASV-GPC was not detectable by plaque assay and only transient and limited replication was confirmed by co-cultivation in Vero and/or by RT/PCR in line with previously published data [189,200,201]. Still, we were able to detect anti-YFV antibodies in YFV17D/LASV∆GPC-immunized animals. Immunization with recombinant YFV17D/LASV∆GPC or combined immunization with YF17D/LASV-GP1 and -GP2 induced the same levels of protection in strain 13 guinea pigs. A single-dose immunization (1x10^5^-1x10^6^ PFU) or prime-boost immunization (on day 14 or 30 with the same doses) and challenge on day 30 after immunization with 1000 PFU of LASV (Jos) protected 80% of strain 13 guinea pigs against fatal LF [144,145]. However, vaccination of common marmosets with recombnant YF17DV expressing LASV∆GPC using the same protocol did not induce protective immune responses. All vaccinated marmosets died with LF clinical manifestations as in control, no vaccine group (I. S. Lukashevich, unpublished). While safety is not a concern for recombinant YFV17D/LAS vaccines, poor immunogenicity would require additional development before it could be a viable option as a bivalent vaccine to control both infections in overlapping endemic areas in Africa.

## 5. Current challenges and problems to overcome

### 5.1. The FDA Animal Rule

The FDA Animal Rule states that under specific circumstances, when human trials would be unethical and unfeasible, the FDA may grant marketing approval to a new vaccine following efficacy trials in adequate and well-controlled animal studies [177]. Whereas pre-clinical toxicity and efficacy studies under the Animal Rule are the same as for clinical trial products, efficacy studies must be performed in more than one well-established and developed animal models. It means to qualify for approval under the Animal Rule, at least two animal models mimicking the pathophysiological mechanisms of human LF must be established in which correlates of protection must be clearly defined.

The NHP are the only relevant model for human LF. Closely-related rhesus (*Macaca mulata*) and cynomolgous (*Macaca fascicularis*) monkeys have been most extensively used for evaluation of promising vaccine candidates or treatment [35,76,77,140,142,202,203,204]. In most of these studies, necropsy was performed at the termination stage of the disease. Nevertheless, recent studies focusing on early stages of the LF in NHP [205] and immune responses [82] confirmed previous observations and reestablish markers of fatal LF: unchecked viremia, elevated liver enzymes, low or undetectable levels of proinflammatory cytokines (IL-1β, TNF-α, IL-8, and IP-10), and low and/or ineffective T cell activation. In addition, high levels of IL-6 was found as an additional biological marker of fatal disease linked to hepatocyte regeneration, which had been previously described in fatally infected LF patients [65] and recently confirmed in our surrogate model of LASV-induced hepatitis in rhesus macaques [80,81]. These studies also showed that early and strong CMI responses were associated with effective control of virus replication and recovery.

Efficacy studies in NHP in BS-4 containment are extremely costly. In addition, there is currently a shortage of rhesus macaques for biomedical research [206]. The development of less expensive and more reliable models of human LF for LASV vaccine research is therefore warranted. The common marmoset, *Callithrix jacchus*, is a small anthropoid primate that generally weighs between 320 to 450 g. The relatively small size of marmosets translates to lower cage and feeding costs, and eases handling in a biosafety environment; these features confer substantial benefits when compared with the use of macaques. Completion of sequence analysis of the entire common marmosets genome will clarify the genetic similarity between the marmosets and humans and will provide access to reliable immunological tools. Because of these advantages, common marmosets have been widely used in many studies involving gene therapy, bacterial infection, toxicology, immunology, and vaccine development [206,207,208,209].

Experimental infection of marmosets with LASV resulted in a systemic disease with high viremia and viral RNA load in tested tissues, elevated liver enzymes, decreased plasma albumin, weight loss, and severe morbidity, the latter which manifests 15 to 20 days after inoculation [84]. Morphological features mirror those described for human cases of fatal LF, and includes hepatic and adrenal necrosis, lymphoid depletion, and interstitial nephritis. Immunochemistry studies of liver and lymphoid tissues revealed marked reduction in CD3+, CD20+ cells, the intensity of HLA-DP, DQ, DR staining, and expression of MHC class II molecules. These observations provided the first experimental evidence that replication of LASV in tissues is associated with immunological alterations that reflect an impaired adaptive immune responses [84]. Common marmosets have been successfully used to evaluate safety, immunogenicity, and efficacy of the LASV ML29 vaccine candidate [143] (see section 4.5.3), providing evidence that LASV infection of marmosets corresponds well with the human LF, like the macaques. Challenge studies in marmosets will considerably reduce cost, especially if breeding colonies are established in-house. LASV infection of marmosets could therefore be the 2nd “small” NHP model to comply with the FDA Animal Rule.

Under current regulation policy, it is highly unlikely that experimental rodents, even the highly susceptible strain 13 guinea pigs, will be considered as an appropriate model for efficacy trials. That is also true for model systems based on infection with host-adapted LASV strains [210] or with less pathogenic “surrogate” arenaviruses (rev. [208]). Recently animal models based on mice with artificially compromised immune system have been described (e.g; mice expressing humanized MHC class I molecules [211], mice deficient for α/β and γ-interferon receptors [212]). These animals can potentially be helpful for basic studies, but probably will not be applicable for efficacy trials.

Although mice do not accurately model human LF disease, they can provide an economical assay to determine vaccine potency via the capacity of vaccine candidates to elicit protective CMI responses. This type of small animal models is especially needed when promising vaccine technology will be transferred from the laboratory to the manufacturing environment. An additional benefit afforded by murine models is a lower level of biocontainment. This level must be lower than BSL-4, because pre-clinical vaccine development in the BSL-4 is not practical. In a recently described CBA/J-ML29 model [178], a T cell cytotoxicity assay *in vivo* showed a correlation between LASV-specific cytotoxicity and protection induced by the LASV vaccine candidate (ML29). Notably, CBA/J mice that received CD8+ T cell-depleted splenocytes from ML29-immunized donors all succumbed to a lethal i.c. challenge, demonstrating that CD8+ T cells are critical in protection. The CBA/J-ML29 model can therefore be a useful immunological tool for evaluation of immunogenicity and efficacy of LASV vaccine candidates outside of BSL-4 containment facilities.

While results of safety and efficacy studies in NHP models must be submitted to FDA, the agency will probably require efficacy trials in humans. The Animal Rule pertains to diseases which are so rare that trials are not feasible (e.g; outbreaks of hemorrhagic fevers caused by EBOV and MARV). In contrast, LF is common disease in West Africa and this argument cannot be made. Indeed, efficacy evaluation of Candid #1 was performed in phase III trial in Argentina [138]. Notably, prevalence of JUNV infection in Argentina is lower than LASV infection in West Africa; 3,500 cases/yr (historical high before introduction of Candid #1) vs. 3,000-5,000/yr cases of LF in West Africa (conservative estimates). Due to strain variations of LASV, a multicenter trial in several endemic areas (countries) would probably have to be conducted to evaluate cross-protective efficacy of vaccine candidates.

### 5.2. Vaccine formulation and target population

Vaccine formulation will probably depend on the vaccination strategy and targeted groups. For general population in LASV endemic areas a single-dose vaccination providing a life-long protection against the disease caused by all genetic clades is the most desirable strategy. A live attenuated vaccine expressing LASV GPC and NP would be an appropriate vaccine candidate to meet this goal. LASV NP is the major component of transcription/replication machinery and this protein is synthesized at early stages of infection, prior to GPC and structural glycoproteins. Thus, effective anti-NP immunity during early stages of the infection will potentially contain and control virus replication. Indeed, mice that were tolerated against all NP-derived T cell epitopes were severely compromised in their ability to control LCMV [213]. These findings are in-line with data from humans, in which asymptomatic, but LASV-seropositive, individuals from endemic areas have very strong memory CD4+ T-cell responses against the NP. Taken together, these findings indicate that anti-NP immunity is involved in efficient virus control and contribute to long-term cross-reactivity [61]. Reassortant ML29 is currently available preventive vaccine candidate inducing sterilizing, cross-protective immunity and acting as an effective post-exposure countermeasure. Recently established LASV reverse genetics systems [158,214] provide powerful tools to insure safety profile and further improvement of this vaccine.

Recombinant VSV∆G-based vaccine candidates that express filovirus glycoproteins were intensively tested in more than 80 NHPs with very encouraging results that provide convincing evidence of the safety of this platform (rev. [150,215]). These results justify further development of VSV∆G as a vaccine platform for human use. Unfortunately, the available information on VSV∆G/LASVGP vaccine is very limited. The mechanism of protection is also not clearly understood and durability of protective immunity is unknown. The cross-protective activity of this vaccine against heterogeneous LASV strains must also be addressed. As a possible solution to this concern, vaccination with a blended vaccine that contains equal amounts of rVSV expressing LASV GPC derived from all LASV clades can be considered.

IMOJEV®, a live-attenuated vaccine obtained by replacing the envelope genes of YF-17D with those of JE is expected to become the first approved human viral vector vaccine [216]. Tetravalent dengue vaccine (Sanofi Pasteur) is currently in phase III of clinical development [217]. The success of YF17D-based vaccine technology provides additional motivation to further develop and improve the YF17D/LASV-GPC vaccine platform. This vaccine can be potentially used as a pediatric bivalent vaccine to control both YF and LF infections in overlapping endemic areas [9]. In spite of positive proof-of-concept results in guinea pigs, the poor immunogenicity of this vaccine failed to protect NHPs. Certainly, additional development efforts are required to improve genetic stability of LASV GP inserts and immunogenicity of this vaccine [128].

The advanced VEEV TC-83 replicon system provides a safe and multivalent solution for making effective LASV vaccine candidates. This platform requires a prime-boost vaccination strategy to achieve desirable levels of immune responses and protection. In practical terms, this strategy would be applicable for “organized” target groups (e.g; first responders, personnel of local hospitals in endemic areas, international travelers visiting endemic areas, military personnel, and staff of BSL4 labs working with LASV). The ability of VEEV RPs to deliver and express at least two foreign genes in target cells [96] provides a good opportunity to make cross-protective vaccines by expressing LASV GPC from different clades and with enhanced immunogenic capacities. 

### 5.3. Conclusions and prospects for future

During the past two decades, biodefense funding has brought new human and financial resources to combat emerging infectious diseases, and resulted in substantial progress in basic research (discovery of new arenaviruses, host cell interaction, reverse genetics, *etc*.). Although progress in vaccine development has been less impressive, at least three vaccine platforms (recombinant vaccinia, MOP/LAS reassortant, and recombinant VSV) provided vaccine candidates that were successful when tested in NHPs. Vaccinia virus is no longer acceptable in Africa with high prevalence of HIV-1, but MOP/LAS reassortant (ML29) and rVSV vaccines have some room for further development, in spite of safety issues surrounding their potential use. The rVSV-based vaccines against filoviruses (EBOV, MARV) have been extensively tested in NHPs with promising results. While filoviruses brought much more public attention and resources from their high mortality and concern over their potential use as bioweapons, they are a relatively minor public health threat for Africa. LASV is a potential bioterrorism threat as well; however, in contrast to filoviruses, LF is also a major public health concern across West Africa [218]. Any progress in clinical development of recombinant VSV-based filovirus vaccines, for example, pre-investigational new drug (IND) approval, will be a step in the right direction [150] and will likely positively impact VSV∆G/LASVGP development as well.

While health authorities of endemic and non-endemic countries have to apply the internationally recognizable regulatory standards to new preventive and therapeutic biologics against emerging infectious threats, it seems that regulatory priorities differ. In the absence of market-driving forces, the approval process in non-endemic countries is very slow. The FDA Animal Rule for regulation of “biodefense” biologics was proposed at the end of the last century and was implemented in 2002. To date, there are no FDA-approved vaccines using the Animal Rule. Meanwhile, in 2007 a live-attenuated vaccine Candid #1, with a clinically proved efficacy of 95% against Argentine hemorrhagic fever , was incorporated in National Immunization Plan in Argentina and tremendously impacted the magnitude of epidemic outbreaks [138,219]. This vaccine was jointly developed by Argentine and US scientists, with support from the national Ministry of Health and US Army Medical Research Institute of Infectious Diseases, under supervision of United Nations Development Program and Pan American Health Organization. In August 2006, after successful clinical trials, the vaccine was registered by the National Regulatory Authorities and is currently manufactured in Argentina. In the U.S. this vaccine has an IND status. It is already made a positive impact on JUNV research. Based and the availability of an effective vaccine, CDC and NIH allowed research with JUNV in BSL3 containment, under the conditions that personnel are vaccinated with Candid #1 [220]. Nevertheless, taking into consideration an apparent negative bias of the FDA regarding replicative-competent vaccines, the feasibility of approval for use in the US is murky. There is a chance that Candid #1 will share the VEEV TC-83 vaccine story. This IND vaccinehas been used for decades to protect military and lab personnel, but it will be never approved by FDA. Nevertheless, so far the Candid #1 story is a promising guide for ML29 vaccine candidate. The major lesson learned from the past is that local needs and responsiveness of national authorities from endemic areas must be important driving forces supported by international development programs and charitable organizations. Recent international initiatives [2] and signs of interest from biotechnology companies [102] provide some hope for the future.
